# Sensory laterality in affiliative interactions in domestic horses and ponies (*Equus caballus*)

**DOI:** 10.1007/s10071-018-1196-9

**Published:** 2018-06-09

**Authors:** Kate Farmer, Konstanze Krüger, Richard W. Byrne, Isabell Marr

**Affiliations:** 1 School of Psychology and Neuroscience, University of St. Andrews, St. Mary’s Quad, South Street, St. Andrews, Fife, Scotland KY16 9JP UK; 2 University of Regensburg, Zoology/Evolutionary Biology, Universitaetsstraße 31, 93053 Regensburg, Germany; 3 Department Equine Economics, Faculty Agriculture, Economics and Management, Nuertingen-Geislingen University, Neckarsteige 6-10, 72622 Nürtingen, Germany

**Keywords:** Laterality, Equine, Affiliative, Behaviour

## Abstract

Many studies have been carried out into both motor and sensory laterality of horses in agonistic and stressful situations. Here we examine sensory laterality in affiliative interactions within four groups of domestic horses and ponies (*N* = 31), living in stable social groups, housed at a single complex close to Vienna, Austria, and demonstrate for the first time a significant population preference for the left side in affiliative approaches and interactions. No effects were observed for gender, rank, sociability, phenotype, group, or age. Our results suggest that right hemisphere specialization in horses is not limited to the processing of stressful or agonistic situations, but rather appears to be the norm for processing in all social interactions, as has been demonstrated in other species including chicks and a range of vertebrates. In domestic horses, hemispheric specialization for sensory input appears not to be based on a designation of positive versus negative, but more on the perceived need to respond quickly and appropriately in any given situation.

**Electronic supplementary material:**

The online version of this article (10.1007/s10071-018-1196-9) contains supplementary material, which is available to authorized users.

## Introduction

Specialization in the functions of the two hemispheres of the brain has been well catalogued and is believed to have its origins in brain asymmetry in early vertebrates (MacNeilage et al. 2009). There is therefore increasing interest in the details of asymmetry of brain function and the different ways in which information is processed and interpreted by each hemisphere. Asymmetry has been observed in many taxa, including mammals, birds, fish, and even insects (for overview see Rogers 2017), and may be expressed as motor laterality (usually limb preference), or sensory laterality (preferential use of a sensory organ on one side of the body). It has been shown that in most situations motor laterality and sensory laterality are not correlated in horses (Austin and Rogers 2012; McGreevy and Rogers 2005), fish (Biazza and Brown 2011; Takeuchi and Hori 2008), new born humans (Cioni and Pellegrinetti 1982), and rhesus monkeys (White et al. 1994).

The close connection between humans and horses as sport and leisure partners makes the understanding of laterality in horses important, as it potentially has wide ranging implications for the welfare and safety of both the horses and the humans. For example, if horses have a preferred side for social interaction, this could be an indication of how training and handling can be carried out most effectively and safely.

Rogers (2004) found that chicks that were not lateralised were slower to respond to a potential predator than lateralised chicks and proposed that lateralisation of the brain may have an evolutionary benefit for animals with side-placed eyes, as it allows for dual attention. This enables, for example, simultaneous attention to be given to foraging and predator vigilance. Additionally, lateralisation may facilitate appropriate reaction to unexpected stimuli as proposed by Austin and Rogers (2007).

To date, research in this field has focussed mainly on aggressive behaviour, stressful situations and negative emotions, in which a preference for left side, and therefore dominance of the right brain hemisphere, has been found consistently. Larose et al. (2006) found emotionality in horses to be linked to using the left eye to observe a novel object, and Austin and Rogers (2007) found stronger reactions to an unexpected stimulus (an opening umbrella) when it was presented on the horse’s left side. Additionally, Smith et al. (2016) observed a left eye bias and an increased heart rate when horses were presented with photographs of an angry-faced human, while Austin and Rogers found a left bias in agonistic and vigilance behaviour in free roaming feral “Brumby” horses (2012) and Przewalski horses (2014). Similar left biases have also been shown in male tree lizards (Hews and Worthington 2001), dogs (Siniscalchi et al. 2010), Australian magpies (Koboroff 2008), and cattle (Robins and Phillips 2010).

In humans, it has been proposed that there is a hemispheric divide in the processing of emotion with the left hemisphere processing positive emotion, and the right hemisphere processing negative emotion (Davidson and Tomarken 1989; Canli et al. 1998; Godfrey and Grimshaw 2016). However, other studies such as Borod et al. (1998) have suggested that the right hemisphere may be used for all emotional processing, and according to Davidson (1992) frontal and anterior areas of the brain differ in the processing of positive and negative emotions, with the right frontal region more strongly active for negative emotions, and posterior regions of the right hemisphere more strongly involved in the perception of positive emotions. Kilgore and Yurgelun-Todd (2007), on the other hand, propose that these various hypotheses may not actually be in opposition, but may instead reflect different facets of a complex distributed emotion processing system.

In non-human primates, numerous studies have shown emotion of all types to be processed in the right hemisphere (overview Lindell 2013), but in horses, while there is a large body of research on laterality in stressful and agonistic situations, there has so far been no dedicated research into sensory laterality in positive interactions between conspecifics. Farmer et al. (2010) observed that domestic horses had a preference to have humans on their left side, and that this preference was stronger in conventionally trained horses, which are handled mostly from the left, than in bilaterally trained horses. Although it cannot be discounted that the presence of the human may have represented a form of stress which could have influenced the lateral preference shown, Karenina et al. (2017) found a left bias in mother–infant interactions across several mammalian species, including horses, which suggests that the right hemisphere is indeed used for positive as well as negative emotions.

Here we examine laterality in affiliative interactions in individuals, comparing groups of different social compositions and breeds. Domestic horses have diverse genealogies and phenotypes and are broadly categorised into (1) race and riding horses, (2) ponies and (3) draught horses (Pirault et al. 2013; Petersen et al. 2013). We therefore considered it possible that the hemispheric specialization might differ between the riding horses and ponies in this study. The groups included a mixed-sex group of riding horses, an all-female group of Mini-Shetland pony mares and foals, a Mini-Shetland pony harem group of one stallion and several mares, and an all-male group of Mini-Shetland pony stallions and colts. The specific questions we addressed were: (1) is sensory laterality in affiliative interactions normally distributed, or is one side preferred over the other; (2) if there is a side preference, is this affected by age, rank, sociability, gender, or phenotype of the individuals; (3) if there is a side preference, is it affected by the social composition of the group?

## Methods, materials, and subjects

### Subjects

Thirty-one privately owned horses and ponies took part in the study, all of them housed at the Aktivstall Mauerbach complex in the Vienna Woods, Austria. There were four groups, each of different social composition.

*Group 1*, riding horses (*N* = 10), comprised 4 geldings and 6 mares, aged from 2 to 22 years. They included 4 Warmbloods, 1 Sorraia Mustang, 1 Pryor Mountain Mustang, 2 Quarter Horses, 1 Icelandic horse, and 1 Haflinger. The group was housed in a “Hit Aktivstall”, designed to cover the needs of horses as well as possible. The stabling covered approximately 2.5 hectares, (2500 m^2^ per horse) and included a rest and sleeping shelter (300 m^2^, enclosed on three sides, with three open doorways on the eastern side). The horses had 24-h access to grass pasture, straw fodder, and water from an automatic dispenser. An automatic group hay feeder opened for 15 min, 16 times per day. There was also an individual automatic hay feeder and an individual automatic pellet feeder, which were programmed according to each horse’s needs, with individual rations varying between 500 g and 2 kg per day. The feeders automatically portioned and dispensed the hay or pellets when activated by a transponder, worn either on a collar around the horse’s neck, or woven into the horse’s mane. To reach the pellet dispenser, the horses had to walk around a track of approximately 700 m. The stabling area also included three grass pastures, covering a total of 2 hectares, which were open to the horses 24 h a day. The horses shared their living quarters with two female donkeys, but as only one interaction was observed between a horse and the donkeys, the donkeys were not considered in the analysis.

*Group 2*, mares and foals (*N* = 8), comprised 5 Mini-Shetland pony mares, 3 with foals at foot. The foals, 2 fillies and 1 colt, were all between 3 and 6 months old at the time of observation, while the mares ranged from 1 to 20 years old. The group was housed on approximately 6400 m^2^ of grass pasture and woodland (800 m^2^ per pony) with two shelters, each of 20 m^2^, which were enclosed on three sides. There was a covered hay station providing ad libitum hay, and water was supplied in large buckets. The area was divided into two grass pastures, a sand enclosure where the hay station was positioned, and an area of woodland which offered shade. The ponies also received approximately 150 g of grain once a day.

*Group 3*, harem (*N* = 8), comprised 1 stallion and 7 mares, all Mini-Shetland ponies, aged 3–14 years old. The group was housed on 0.7 hectares of mixed grass pasture and woodland, (970 m^2^ per pony). Hay was provided in hanging dispensers and nets, as well as in fixed stands. Fresh water was available from a stream, as well as in large buckets. The ponies also received approximately 150 g of grain once a day. There were two shelters, each enclosed on three sides: one of 72 m^2^, one of 48 m^2^.

The stallion was removed from the group for management purposes the evening before the final observation period; however, as the absence of a stallion has been shown to slightly increase social interaction in mares (Sigurjónsdóttir et al. 2003), we continued to collect data on the mares.

*Group 4*, stallions (*N* = 5), comprised 3 mature stallions and 2 yearling colts, all Mini-Shetlands, aged from 1 to 20 years old. The group was housed on a 2-hectare grass pasture (4000 m^2^ per pony), with shade provided by trees along one side and a small grove in the centre. There were two shelters each measuring 48 m^2^ and enclosed on three sides. The ponies shared this pasture with nine sheep, but there was very little contact between the sheep and the ponies. The grass was so plentiful that additional hay was not considered necessary, but the ponies did receive approximately 150 g of grain once a day. Water was supplied in buckets and automatic drinkers.

### Observation

Groups 1, 2 and 3 were observed for 12 h each, and group 4 (which had fewer individuals) for 10 h, between July 4th and July 21st, 2017. Observation was carried out between 10 a.m. and 6 p.m., in periods of between 1.5 and 2.5 h. The observation periods for each group were randomised across the times of day, and no group was observed more than once on any 1 day. Observer 1 (KF) recorded each observation verbally on the voice recorder of a Samsung A3 mobile phone, and Volunteer 1 made video recordings of the observations on an iPhone6 as a backup and cross reference. The data from the recordings were transferred to an Excel 2013 sheet on a Packard Bell “Easy Note” laptop immediately after the observation period. The recordings and data sheets were then backed up on USB sticks. Volunteer 2 transcribed the voice recordings into text.

All the horses and ponies were already acclimatised to the presence of people, and the observation points were based between 10 and 30 m away from each group, although the precise distance depended on the movement of the horses/ponies, and whether the observers had to move in closer to see the details of an interaction. There was no point at which the horses and ponies appeared to be disturbed by the presence of the observers. When horses or ponies spontaneously approached the observers, they were gently encouraged to move away and return to other members of their group.

Volunteer 1 simply recorded the video and did not make any rating or comment and so could not be used for an inter-observer rating. Therefore, a sample of 10% of the videos was shown to volunteer 3, who made an independent assessment of the behaviour. There was a high level of agreement between observer 1 and volunteer 3, with a Cohen’s Kappa coefficient of *k* = 0.932.

### Data collection

#### Affiliative interactions: approaches and interactions

We defined affiliative approaches by considering the behaviour of the approached horse. If the approached horse retreated more than two metres from the approaching horse, the approach was considered non-affiliative. If the approached horse did not move, moved towards the approaching horse, or moved less than 2 m to make room for the approaching horse, the approach was considered affiliative, as described by Schneider and Krüger (2012). Affiliative interactions typically included allo-grooming, swishing flies for each other, and standing in a proximity of less than 2 m for at least 15 s while grazing or resting. The side placement of equine eyes makes it easy to see whether one eye or the other is being preferred in any interaction. One horse approaching another with its left eye to the approached horse scored one point under “affiliative left” for the approach, or “affiliative right” if the approach was with the right eye. A further point was allocated both to the approaching and to the approached horse if the approach led to allo-grooming, nose to tail fly swishing, or just relaxing and standing within 2 m of each other for at least 15 s, according to the side of the interaction.

If a pair of horses switched sides, further points were allocated to each horse accordingly, and if a pair positioned themselves side by side, affiliative points were awarded to each horse according to the eye used for viewing the conspecific.

Interactions where a lateral choice could not be established (for example, a head-on approach) were not scored for the sensory laterality data but were included in the rank dominance calculations if appropriate. As head-on approaches only occurred in agonistic encounters, these were excluded from the affiliative laterality analysis.

#### Rank dominance observations

Rank dominance points were awarded based on retreats by either the approaching or the approached horse. The retreating horse was allocated one point under “lose” for a retreat, and the horse that was retreated from was awarded a point under “win”. Non-affiliative interactions were defined as approaches with the ears pinned back and the nose extended, retreats, threats to bite or kick, bites, kicks and chases as described by McDonnell and Haviland 1995, and McDonnell 2003.

Approaches and interactions were scored under the categories (1) affiliative left, (2) affiliative right, (3) win, and (4) lose.

### Data and statistical analysis

Affiliative laterality index (ALI): an ALI was calculated for each horse, using the standard formula of (right eye score − left eye score)/total lateral interactions, as used by Austin and Rogers (2012). This gives scores between − 1 and + 1 with negative scores showing a left bias, and positive scores a right bias.

*Social index (SI)* an index was calculated for each horse using all interactions, where the SI = (affiliative interactions − non-affiliative interactions)/total interactions. This gives a number from − 1 to + 1, with positive numbers indicating relatively more affiliative behaviour.

*Dominance Index* An average dominance index (ADI) was calculated as recommended by Hemelrijk et al. (2005). ADI = 1/*N* ∑_*j*_[*x*_*ij*_/(*x*_*ij*_ + *x*_*ji*_)]; *N* the number of interaction partners, *x*_*ij*_ the number of times the individual *i* won against conspecific *j, x*_*ji*_ the number of times individual *i* lost against conspecific *j*. ADI values range from 0 to 1, with a high value indicating a high rank in the group. Individuals were counted as a winner when their interaction partner retreated one step or more. Pairs that were not involved in an encounter with each other were excluded from the analysis.

The R Studio and R commander (version 3.4.1, 2017) were used to analyse the data and compare the laterality indices across groups, gender, rank, and social index. Figures and tables were compiled in Microsoft Excel 2016.

The ALI was not normally distributed (Shapiro Wilk test). We therefore continued using non-parametric tests. We considered the numbers of literately indices to the left and the right for each individual and used a binomial test to analyse the level of bias on population and individual levels. Multivariate factor analysis [GLM, formula = ALI ~ age + phenotype + gender + group + rank + social index, family = Gaussian (identity)] was used to compare the four groups with respect to the variables of phenotype and group composition, and to compare the variables of age, gender, social index, and rank within the groups. All the tests used were two sided and the significance level was set at 0.05.

## Results

A total of 2475 interactions (2043 affiliative and 432 non-affiliative) were recorded among the 31 horses and ponies. Details of the interactions and categorizations are shown in Table 1, and the raw data table is included in the supplementary material.

**Table 1:** Raw data collected in July 2017 from horses and ponies at Aktivstall Mauerbach, Austria

Horse ID and gender	Age	Gender	Rank index	Social index	Total affiliative approaches and interactions	Left side affiliative approaches and interactions	Affiliative laterality index	*p < 0.05, **p < 0.01
Group 1: riding horses								
Alia	13	Mare	0.86	− 0.17	22	12	− 0.09	
Amaluna	2	Mare	0.17	0.89	102	55	− 0.08	
Annie	19	Mare	0.32	0.76	87	58	− **0.33**	**
Bayladora	6	Mare	0.45	0.26	29	21	− **0.45**	*
Baika	22	Mare	0.55	− 0.30	31	21	− **0.35**	*
Billy	14	Gelding	0.83	− 0.45	16	10	− 0.25	
Eco	11	Gelding	0.95	0.45	103	58	− 0.13	
Kyakur	15	Gelding	0.25	0.87	119	64	− 0.08	
Moon	16	Gelding	0.37	0.75	48	29	− 0.21	
Sharon	2	Mare	0.02	0.94	96	61	− **0.27**	**
Group 2: mini-pony mares and foals								
Zenith	11	Mare	0.93	0.75	47	27	− 0.15	
Cioca Tino	6 months	Colt	0.25	0.96	102	59	− 0.16	
Magreeth	20	Mare	1.00	0.66	29	14	0.03	
Cinne Bun	3 months	Filly	0.22	0.97	134	83	− **0.24**	**
Sita	15	Mare	0.59	0.68	48	26	− 0.08	
Buttercup	4 months	Filly	0.47	0.92	136	82	− **0.21**	*
Tiramisu	1	Mare	0.14	0.78	41	25	− 0.23	
Sara Jane	7	Mare	0.65	0.56	21	11	− 0.05	
Group 3: mini-pony harem								
Versace	9	Stallion	0.85	0.35	21	12	− 0.14	
Sun Suena	3	Mare	0.26	0.67	61	34	− 0.11	
Funny Honey	3	Mare	0.19	0.80	80	50	− **0.25**	*
Andromeda	3	Mare	0.72	0.24	50	28	− 0.12	
Dusky	3	Mare	0.29	0.64	54	32	− 0.19	
Blissful	3	Mare	0.08	0.88	47	27	− 0.15	
Goldie	7	Mare	0.81	0.28	66	41	− **0.24**	*
Mascara	14	Mare	0.88	0.27	54	32	− 0.19	
Group 4: mini-pony stallions and colts								
Horatio	20	Stallion	0.40	0.90	59	32	− 0.08	
Versace	9	Stallion	0.90	0.82	71	46	− **0.30**	**
Amasonic	10	Stallion	0.83	0.85	63	28	− 0.11	
Frappuccino	1	Colt	0.11	0.98	100	49	− 0.02	
Toffee Popcorn	1	Colt	0.00	1.00	106	57	− 0.08	

Bold type shows significant values

The ALI values within each group were independent of age, sex, rank, social index, phenotype, and group composition (GLM: *N* = 31, all *p* > 0.05). However, there was a weak trend for the riding horses to be more strongly lateralised (Wilcoxon rank sum test: *N* = 31, *W* = 65, *p* = 0.09) and this is illustrated in Fig. 2 in the supplementary material.

**Fig. 1:**
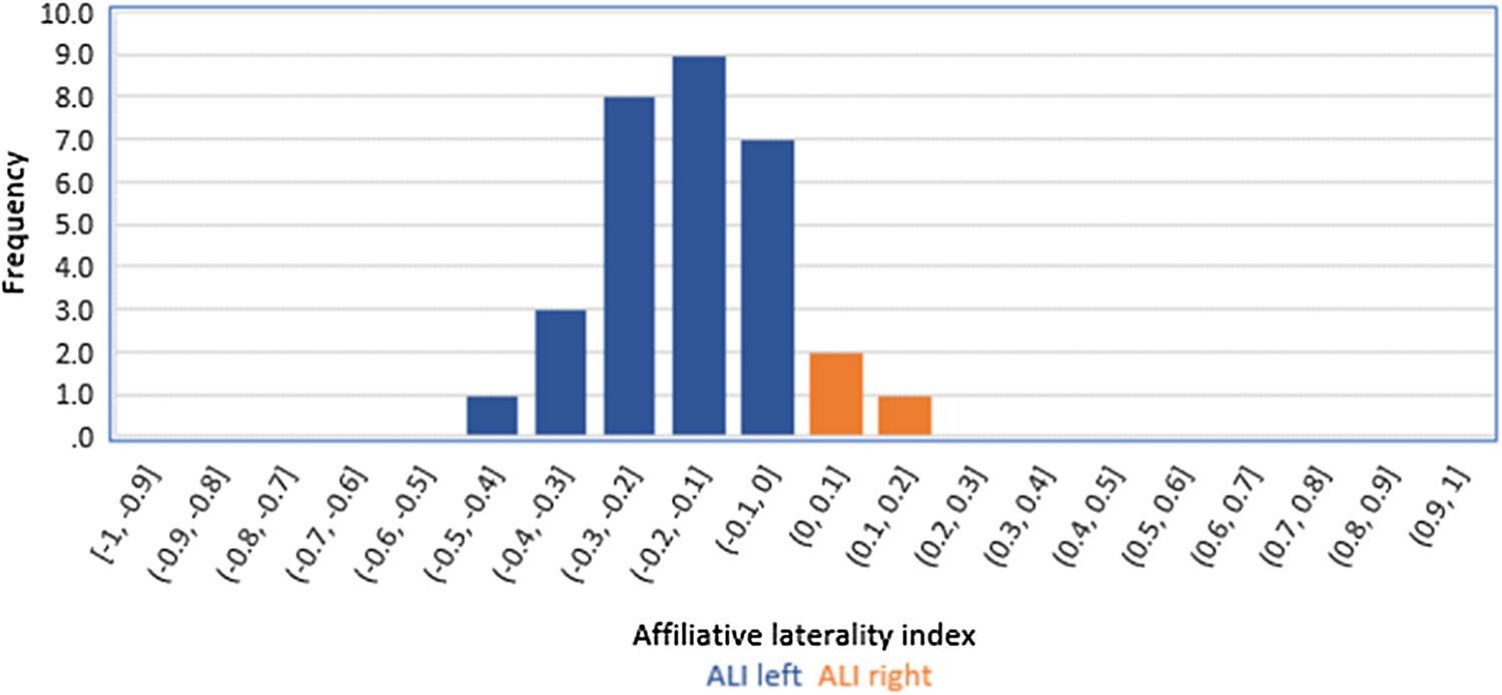
Distribution of affiliative laterality indices showing a clear bias to the left

A binomial test indicated that the proportion of animals showing a left bias of 0.9 was higher than the expected 0.5 (binomial test, two sided: *N* = 31, *p* < 0.001). Additionally, 4 horses and 5 ponies, showed significant individual left preferences in their affiliative interactions (binomial test, two sided: all *p* < 0.05). See Fig. 1.

## Discussion

Our results do not support the hypothesis that lateral choices in affiliative interactions are normally distributed, but instead indicate a consistent and significant bias to the left. We found no evidence that this left bias is affected in direction or strength by age, rank, sociability, phenotype, or sex. The weak trend for the riding horses to be more strongly lateralised than the ponies was not significant, but further research with larger sample sizes is required to investigate this more thoroughly. McGreevy and Thompson (2006) found that motor laterality varied according to breed in performance horses, and Larose et al. (2006) found that a more emotional breed of horse (French Saddlebred) showed stronger sensory laterality in a novel object test than a more phlegmatic breed (Trotter), so it is certainly possible that sensory laterality in affiliative interactions may vary also according to breed and type.

Interestingly, the strength and distribution of the left bias we observed in the horses’ affiliative interactions corresponds very closely to the left biases in agonistic and vigilance behaviour observed by Austin and Rogers (2012, 2014) in feral and Przewalski horses. This supports the theory that the right hemisphere is preferred for the processing of both positive and negative emotions as proposed by Davidson (1992).

Numerous studies have found that allo-grooming, and even grooming by humans, can significantly reduce a horse’s heart rate (e.g. Feh and Mazières 1993; Normano et al. 2003), and activities such as allo-grooming and swishing flies do not appear to be stressful (Feh and Mazières 1993). These interactions are shown in this study to be lateralised to the left, as has been shown in comparable interactions in fish (Sovrano et al. 1999), chicks (Vallortigara and Andrew 1994) and numerous vertebrates (Karenina et al. 2017). This again suggests that the right hemisphere may specialise in processing social interactions and emotions, both positive and negative. In fact, de Boyer des Roches et al. (2008) reported that horses preferred to use their left eye to observe an object with a negative emotional association (a vet’s jacket) and for an object with positive association (a feed bucket), while the right eye was preferred for a neutral object (a traffic cone).

Rogers (2017) proposes that the strength of laterality is of greater significance than the direction, and it has been shown that laterality increases with the level of concentration and task complexity in vervet monkeys (Harrison and Byrne 2000). It is therefore possible that the observed laterality in affiliative interactions is simply an indicator of how much attention the horse or pony is applying, and how much emotional involvement it is experiencing. It is not necessarily an indicator of the nature of the attention or emotion, or whether the horse or pony is experiencing stress or eustress. Further research is needed into the factors that may influence the strength of sensory laterality in affiliative behaviour, and into the influence of specific breeding and training. This may then prove to be useful, together with physiological parameters, in the assessment of animal welfare.

## Conclusion

Based on the sample of 31 riding horses and Mini-Shetland ponies, this study shows for the first time that affiliative behaviour in horses and ponies is significantly left lateralised. This adds a new dimension to research into sensory laterality in equids which has, to date, focused on agonistic encounters, which also show a left bias. The fact that there is now evidence that processing of all social interactions is left lateralised means that, in practical and welfare terms, a low level of left laterality is to be expected and does not have implications as to whether a particular experience is positive or negative. The bias for social processing on the left is consistent with the traditional belief that new tasks should usually be taught from the left before transferring to the right. Recognising the horse’s preference in this could potentially reduce stress and make training safer and more successful. A further study on a larger number of animals, including equines of different breeds and types, and under different types of human management and training, is needed to investigate this in detail.

## Electronic supplementary material

Below is the link to the electronic supplementary material.

Supplementary material 1 (XLSX 44 KB)

Supplementary material 2 (DOCX 14 KB)

Supplementary material 3 (PDF 58 KB)

Supplementary material 4 (M4V 11457 KB)

Supplementary material 5 (M4V 13722 KB)
