# Supplementary material for: Sensory laterality in affiliative interactions in domestic horses and ponies (Equus caballus)
Source: Anim Cogn. 2018 Jun 9;21(5):631–7. doi: 10.1007/s10071-018-1196-9 (PMC6097077; doi:10.1007/s10071-018-1196-9)
Supplement: Supplementary file 2 — Supplementary material 2 (DOCX 14 KB) [file 10071_2018_1196_MOESM2_ESM.docx]

**Figure 2: Distribution of ALIs showing the trend for domestic horses to be slightly more strongly left lateralised than Mini-Shetland ponies**
