# Supplementary material for: Sensory laterality in affiliative interactions in domestic horses and ponies (Equus caballus)
Source: Anim Cogn. 2018 Jun 9;21(5):631–7. doi: 10.1007/s10071-018-1196-9 (PMC6097077; doi:10.1007/s10071-018-1196-9)
Supplement: Supplementary file 3 — Supplementary material 3 (PDF 58 KB) [file 10071_2018_1196_MOESM3_ESM.pdf]

Supplementary material: Details of statistical analysis performed in R Studio and R commander (version 3.4.1, 2017)

```
Call:
glm(formula = friendly_lat_index ~ age + domestication_num + gender_num +
group_num + rank + social_index, family = gaussian(identity),
    data = Dataset)

Deviance Residuals:
     Min        1Q    Median        3Q       Max
-0.25847  -0.05255  -0.01936   0.06754   0.17684

Coefficients:
                   Estimate Std. Error t value Pr(>|t|)
(Intercept)       -0.457774   0.128112  -3.573  0.00154 **
age                0.001554   0.003878   0.401  0.69219
domestication_num  0.053735   0.095284   0.564  0.57802
gender_num         0.050475   0.052285   0.965  0.34398
group_num          0.007565   0.037812   0.200  0.84312
rank               0.088735   0.095671   0.928  0.36290
social_index       0.102087   0.076831   1.329  0.19644
---
Signif. codes:  0 '***' 0.001 '**' 0.01 '*' 0.05 '.' 0.1 ' ' 1

(Dispersion parameter for gaussian family taken to be 0.01225402)

    Null deviance: 0.41659  on 30  degrees of freedom
Residual deviance: 0.29410  on 24  degrees of freedom
AIC: -40.419

Number of Fisher Scoring iterations: 2
```

Spearman correlations:

```
                      age friendly_lat_index
age                1.0000             0.0517
friendly_lat_index 0.0517             1.0000

 Number of observations: 31

 Pairwise two-sided p-values:
                   age    friendly_lat_index
age                       0.7824
friendly_lat_index 0.7824
```

Wilcoxon rank sum test with continuity correction

```
data:  friendly_lat_index by domestication
W = 65, p-value = 0.09425
alternative hypothesis: true location shift is not equal to 0

RcmdrMsg: [6] WARNUNG: Warning in wilcox.test.default(x = c(-0.09, -0.08, -
0.33,
RcmdrMsg+ -0.45, -0.35, :
RcmdrMsg+ cannot compute exact p-value with ties
```
